# Supplementary material for: Multi‐dataset identification of innovative feature genes and molecular mechanisms in keratoconus
Source: J Cell Mol Med. 2024 Sep 19;28(18):e70079. doi: 10.1111/jcmm.70079 (PMC11412914; doi:10.1111/jcmm.70079)
Supplement: Supplementary file 2 — Material S2. [file JCMM-28-e70079-s002.zip › 7.GSEA analysis/3.DBNDD1/KEGG_GLYCOSAMINOGLYCAN_BIOSYNTHESIS_KERATAN_SULFATE.html]

Details for gene set KEGG\_GLYCOSAMINOGLYCAN\_BIOSYNTHESIS\_KERATAN\_SULFATE[GSEA]

|  || Dataset | DBNDD1.group.cls#h\_versus\_l |
| Phenotype | group.cls#h\_versus\_l |
| Upregulated in class | l |
| GeneSet | KEGG\_GLYCOSAMINOGLYCAN\_BIOSYNTHESIS\_KERATAN\_SULFATE |
| Enrichment Score (ES) | -0.4522874 |
| Normalized Enrichment Score (NES) | -1.3000957 |
| Nominal p-value | 0.20303605 |
| FDR q-value | 0.7540518 |
| FWER p-Value | 0.961 |
Table: GSEA Results Summary

  

Fig 1: Enrichment plot: KEGG\_GLYCOSAMINOGLYCAN\_BIOSYNTHESIS\_KERATAN\_SULFATE      
 Profile of the Running ES Score & Positions of GeneSet Members on the Rank Ordered List

  

| SYMBOL | TITLE | RANK IN GENE LIST | RANK METRIC SCORE | RUNNING ES | CORE ENRICHMENT || 1 | CHST2 | na | 2013 | 0.299 | 0.0934 | No |
| 2 | CHST6 | na | 9147 | 0.083 | -0.0698 | No |
| 3 | B4GALT2 | na | 10143 | 0.070 | -0.0632 | No |
| 4 | B4GAT1 | na | 11217 | 0.056 | -0.0659 | No |
| 5 | B3GNT7 | na | 16355 | 0.013 | -0.2071 | No |
| 6 | ST3GAL1 | na | 17755 | 0.005 | -0.2451 | No |
| 7 | B4GALT4 | na | 20368 | -0.016 | -0.3121 | No |
| 8 | B3GNT2 | na | 23789 | -0.053 | -0.3835 | Yes |
| 9 | B4GALT1 | na | 23830 | -0.054 | -0.3573 | Yes |
| 10 | B4GALT3 | na | 25120 | -0.072 | -0.3577 | Yes |
| 11 | CHST4 | na | 28405 | -0.141 | -0.3809 | Yes |
| 12 | FUT8 | na | 30819 | -0.215 | -0.3417 | Yes |
| 13 | CHST1 | na | 31307 | -0.233 | -0.2376 | Yes |
| 14 | ST3GAL2 | na | 32559 | -0.298 | -0.1227 | Yes |
| 15 | ST3GAL3 | na | 33440 | -0.365 | 0.0371 | Yes |
Table: GSEA details [plain text format]

  

Fig 2: KEGG\_GLYCOSAMINOGLYCAN\_BIOSYNTHESIS\_KERATAN\_SULFATE      
 Blue-Pink O' Gram in the Space of the Analyzed GeneSet

  

Fig 3: KEGG\_GLYCOSAMINOGLYCAN\_BIOSYNTHESIS\_KERATAN\_SULFATE: Random ES distribution      
 Gene set null distribution of ES for **KEGG\_GLYCOSAMINOGLYCAN\_BIOSYNTHESIS\_KERATAN\_SULFATE**
